# Proteomic analysis of sputum reveals novel biomarkers for various presentations of asthma

**DOI:** 10.1186/s12967-017-1264-y

**Published:** 2017-08-04

**Authors:** Chao Cao, Wen Li, Wen Hua, Fugui Yan, Hao Zhang, Huaqiong Huang, Yinghua Ying, Na Li, Fen Lan, Shaobin Wang, Xiao Chen, Jing Li, Jinkai Liu, Tianwen Lai, Zhengqiang Bao, Yuan Cao, Yun Zhao, Gang Huang, Lili Huang, Yaqing Huang, Ping Wu, Chao Peng, Zhihua Chen, Kian Fan Chung, Nanshan Zhong, Songmin Ying, Huahao Shen

**Affiliations:** 1 Department of Respiratory and Critical Care Medicine, Second Affiliated Hospital, Zhejiang University School of Medicine, Hangzhou, China; 2 State Key Lab. for Respiratory Diseases, The First Affiliated Hospital, Guangzhou Medical University, Guangzhou, China; 3 National Center for Protein Science Shanghai, Institute of Biochemistry and Cell Biology, Shanghai Institutes for Biological Sciences, Chinese Academy of Sciences, Shanghai, China; 4 Shanghai Science Research Center, Chinese Academy of Sciences, Shanghai, China; 5 National Heart & Lung Institute, Imperial College, London, UK; 6 Department of Pharmacology, Zhejiang University School of Medicine, Hangzhou, China

**Keywords:** Classic asthma, Cough variant asthma, Chest tightness variant asthma, Clinical characteristics, Airway inflammation, Proteomic characteristics

## Abstract

**Background:**

It is now recognized that asthma can present in different forms. Typically, asthma present with symptoms of wheeze, breathlessness and cough. Atypical forms of asthma such as cough variant asthma (CVA) or chest tightness variant asthma (CTVA) do not wheeze. We hypothesize that these different forms of asthma may have distinctive cellular and molecular features.

**Methods:**

30 patients with typical or classical asthma (CA), 27 patients with CVA, 30 patients with CTVA, and 30 healthy control adults were enrolled in this prospective study. We measured serum IgE, lung function, sputum eosinophils, nitric oxide in exhaled breath (FeNO). We performed proteomic analysis of induced-sputum supernatants by mass spectrometry.

**Results:**

There were no significant differences in atopy and FEV_1_ among patients with CA, CVA, and CTVA. Serum IgE, sputum eosinophil percentages, FeNO, anxiety and depression scores were significantly increased in the three presentations of asthmatic patients as compared with healthy controls but there was no difference between the asthmatic groups. Comprehensive mass spectrometric analysis revealed more than a thousand proteins in the sputum from patients with CA, CVA, and CTVA, among which 23 secreted proteins were higher in patients than that in controls.

**Conclusions:**

Patients with CA, CVA, or CTVA share common clinical characteristics of eosinophilic airway inflammation. And more importantly, their sputum samples were composed with common factors with minor distinctions. These findings support the concept that these three different presentations of asthma have similar pathogenetic mechanism in terms of an enhanced Th2 associated with eosinophilia. In addition, this study identified a pool of novel biomarkers for diagnosis of asthma and to label its subtypes.

*Trial registration* http://www.chictr.org.cn (ChiCTR-OOC-15006221)

**Electronic supplementary material:**

The online version of this article (doi:10.1186/s12967-017-1264-y) contains supplementary material, which is available to authorized users.

## Background

Asthma is a common and complex disorder defined as reversible airflow limitation or bronchial hyperresponsiveness with appropriate clinical symptoms [1]. Typical or classic asthma (CA), which presented with wheezing, accompanied with or without dyspnea, cough, or chest tightness, was very well understood and frequently diagnosed. However, there are groups of patients, whose presenting symptoms were not typical wheezing, show airways hyper-responsiveness and excellent response to bronchodilators treatment.

It has been proposed since decades ago that asthma might be presented with atypical symptoms, without obvious wheezing [2]. There is evidence that asthma can present solely with cough, shortness of breath, chest tightness [3, 4]. Recent accumulating evidence suggested that asthma is a pronounced heterogeneity chronic disease with respect to age of onset, clinical characteristics, and response to therapeutics [5, 6]. Clinical heterogeneity of the presence of several disease subtypes may imply a distinct functional or pathobiological mechanism [7].

In 1979, Corrao et al. [8] firstly described a group of patients as cough-variant asthma (CVA) for whom cough was a sole presenting symptom. In 2013, we reported a new clinical variant of asthma: chest tightness variant asthma (CTVA) with chest tightness as a sole presenting symptom [9]. Clinical diagnosis of CVA or CTVA can be made when cough or chest tightness is the only symptom associated with airway hyper-responsiveness with a therapeutic response to asthma therapy.

Because wheezing has long been considered the *sine qua non* of asthma, CVA and CTVA have often been under-diagnosed or mis-diagnosed for their lack of wheezing, and because of poor understanding of its clinical characteristics, and lack of functional biomarkers. Although it has been nearly 40 years since the initial proposal of the pronounced heterogeneity of asthma, it remains unclear whether atypical asthma is representative of an early form of asthma. We hypothesized that different presentations of asthma might possess distinctive cellular and molecular features. This study is designed to identify similarity and to compare differences between different presentations of asthma at various levels.

In an attempt to address these key questions, we analyzed history of atopy, serum immunoglobulin E (IgE), lung function, sputum eosinophil counts, FeNO, anxiety and depression scores in patients with CA, CVA, or CTVA, in comparison with healthy controls. We further performed a proteomic analysis of the sputum supernatants from these patients with CA, CVA, or CTVA to determine whether these profiles would be different.

## Methods

### Study subjects

Classic asthma, CVA, and CTVA subjects were recruited from the Department of Respiratory and Critical Care Medicine, Second Affiliated Hospital of Zhejiang University School of Medicine, Hangzhou, China. Thirty sex-, age-, and ethnic-matched healthy control subjects were enrolled from the community. All participants gave written informed consent to participate in the study, which was approved by the Institutional Review Board for Human Studies of Second Affiliated Hospital of Zhejiang University School of Medicine (Hangzhou, China). The definition of health controls and patients with asthma was according to Global Initiative for Asthma (GINA) guidelines and relevant references [8–11]. A diagnosis of asthma was accepted based on relevant symptoms (cough and chest tightness should be the sole symptom for CVA and CTVA, respectively) and at least one of the following criteria: (1) a 12% and greater than 200-mL FEV_1_ increase after inhaling 400 µg salbutamol; (2) a positive bronchial challenge test; (3) variability in diurnal peak expiratory flow (PEF) of more than 10% for 1 day during 1 week. For CVA, cough should be lasting more than 8 weeks. Exclusion criteria for enrollment included respiratory tract infection in the preceding 8 weeks, other chronic pulmonary diseases, history of drug or alcohol abuse or with a history of mental illness, obvious abnormal of chest HRCT, other pulmonary disease, cardiovascular diseases, significant comorbidity likely to influence the conduct of the study, pregnancy, and breast-feeding.

### Fractional exhaled nitric-oxide (FeNO) measurement

FeNO was measured using a chemiluminescence analyzer (NiOX MINO; Aerocrine, Stockholm, Sweden) at a flow rate of 50 mL/s, according to the the American Thoracic Society/European Respiratory Society guidelines [12].

### Sputum induction and processing

Sputum induction was performed according to previous study [13]. Briefly, sputum was induced with an ultrasonic wave nebulizer. Hypertonic saline in concentrations of 3% was inhaled for 15–30 min, and sputum was attempted sampled after inhalation. For differential cell count, sputum samples were dispersed using four times the weight of phosphate-buffered saline (PBS) containing 0.1% ditriothreitol (Sigma, St Louis, MO, USA). Sputum samples were agitated with a vortex for 5–10 s, and then treated in water baths at 37 °C for 10 min, filtered through a filtered through a 50 µm nylon filter, and centrifuged at 3000 rpm for 10 min at 4 °C. The supernatants were stored at −80 °C and the cells were stained using the hematoxylin–eosin staining for differential cell counting.

### Protein measurements in induced sputum

Protein concentrations of sputum samples were estimated using a BCA Protein Assay Kit (Pierce, Rockford, IL). Proteins (200 μg) from individual samples were extracted by acetone sedimentation. Acetone-precipitated samples were dissolved in 8 M urea, 100 mM Tris–HCl (pH 8.5). Proteins were reduced with 5 mM Tris-(2-carboxyethyl) phosphine (TCEP) at room temperature for 20 min. Alkylation was performed by the addition of 10 mM iodoacetamide, and the samples were incubated at room temperature for 15 min. The protein mixture was diluted four times and tyrptic digested with Trypsin at 1:100 (w/w) (Promega, http://www.promega.com/). Tryptic digests of proteins were analysed by reverse-phase HPLC/tandem MS (MS/MS). The samples were desalted by spin column (Pierce, 89870), and then analyzed by an in-house packed reversed-phase C18 column (360 μm OD × 75 μm ID) connected to an Easy-nLC 1000 HPLC system by a 3 h-gradient at a flow rate of 300 nL/min. The eluted peptides were ionized and introduced into an Orbitrap Elite mass spectrometry (Thermo Orbitap) using a nanospray source. The identity of the compound and its molecular mass from m/z 300–1800 were acquired by the precursor ion scan using the Orbitrap analyzer with resolution r = 60,000 at m/z 400, followed by 20 MS/MS events in LTQ velos analysis. The top 20 most intense precursor ions in each MS scan were sequentially isolated and fragmented with a normalized energy of 35% of CID.

### Statistical analysis

Data were entered into SPSS for Windows, version 13.0 (SPSS Inc., Chicago, IL, USA). The demographic and clinical data were presented as mean ± SD or number (percentage). Serum total IgE values were log-transformed before analysis and expressed as a geometric mean with a range of 1 SD. Categorical variables were assessed using Pearson’s Chi square tests. Comparisons of continuous data between two groups were performed by *t*-test. Comparisons of continuous data among three groups were performed by ANOVA test. The multi-omics data analysis tool, OmicsBean, was used to analyze the obtained proteomics data (http://www.omicsbean.com:88/). In order to improve reliability and interpretability, data were log transformed and normalized by MetaboAnalyst 2.0 [14]. MetaboAnalyst 2.0 provides 11 different procedures for data transformation and normalization and can predict which normalization step is most appropriate for a given data set. Principal components analysis (PCA) of protein expression was used to assess the major sources of variation among samples. PCA reduces the complexity of a multidimensional analysis into two principal components, PC1 and PC2, which orthogonally divide the samples based on the two largest sources of variation in the dataset. In addition, partial least squares discrimination analysis (PLS-DA) was further utilized to quantitatively examine the separability of full spectrum using full-cross validation strategy. The relationship between eosinophils and the levels of proteins was assessed by Pearson correlation. In each analysis, a P-value <0.05 was taken to indicate statistical significance.

## Results

### Demographic characteristics

**Fig. 1:**
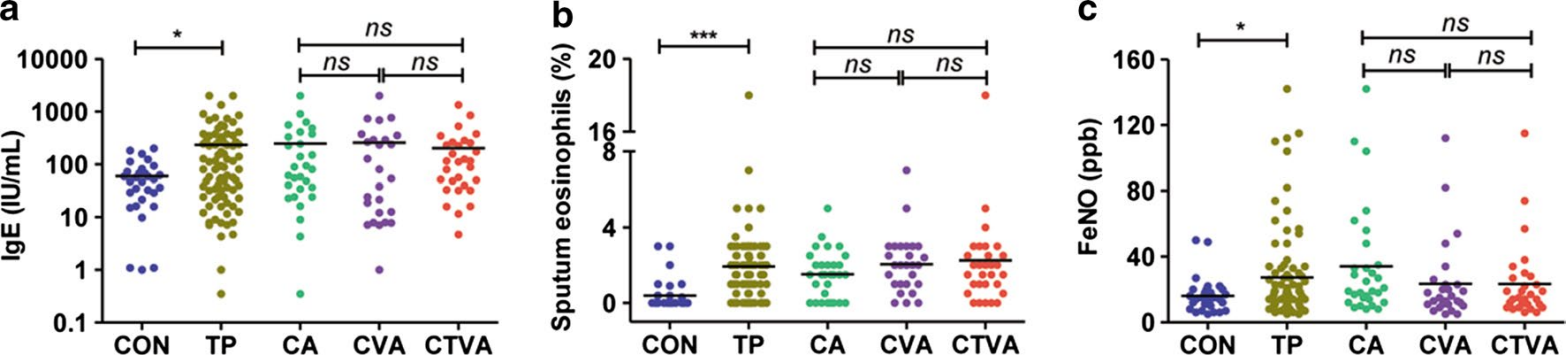
Serum IgE (**a**), Sputum Eosinophil percentages (**b**), and FeNO (**c**) in health controls and asthma patients. Mean values are represented as horizontal bars. *P < 0.05, **P < 0.01, ***P < 0.001, *ns* not significant, *TP* total patients, *CA* classic asthma, *CVA* cough-variant asthma, *CTVA* chest tightness variant asthma, *FeNO* fractional exhaled nitric-oxide

Basic demographic characteristics for health controls and patients with all three asthmatic groups are summarized in Table 1. There was no significant difference in age, gender, and body mass index (BMI) between control and total asthma patients. 30 patients with CA, 27 patients with CVA, and 30 patients with CTVA were enrolled in the study of clinical characteristics. The three asthmatic groups showed no significant differences with respect to age, sex, or BMI. Not surprisingly, patients included in our study had a higher serum IgE levels (*P* < 0.001) (Fig. 1a), increased levels of eosinophil in induced sputum (*P* < 0.001) (Fig. 1b), and FeNO (*P* = 0.002) (Fig. 1c), as these were known factors in patients with asthma. In addition, patients with asthma showed a higher Self-rating Anxiety Scale (SAS) (*P* < 0.001) and Self-rating Depression Scale (SDS) (*P* < 0.001) than those of controls.

**Table 1:** Demographic and clinical features of included subjects

	Healthy controls	Patients with asthma				*P*_Controls vs. Patients_	*P*_CA vs. CVA vs. CVTA_
		Total	CA	CVA	CTVA		
No. of patients	30	87	30	27	30		
Age, years	44.0 ± 12.8	42.1 ± 13.8	41.4 ± 13.6	45.0 ± 13.2	40.1 ± 14.4	0.501	0.399
Male sex, no. (%)	15 (50)	42 (48)	14 (47)	10 (37)	18 (60)	0.871	0.218
Smoking status							
Smokers, no. (%)	9 (30)	13 (17)	3 (10)	4 (15)	6 (20)	0.069	0.554
Pack/years	33.1 ± 42.0	20.5 ± 18.9	21.6 ± 25.0	22.5 ± 20.6	21.5 ± 17.4	0.322	0.997
History of atopy, no. (%)	6 (20)	30 (34)	13 (43)	10 (37)	7 (23)	0.138	0.250
BMI, kg/m^2^	24.3 ± 3.7	23.2 ± 2.8	23.6 ± 2.4	23.7 ± 2.8	22.5 ± 3.1	0.085	0.187
Serum IgE, IU/mL	1.5 ± 0.6	1.9 ± 0.7	1.9 ± 0.8	1.8 ± 0.8	2.0 ± 0.6	0.014	0.668
FEV_1_, %Predicted	99.1 ± 13.5	85.9 ± 16.1	80.7 ± 14.6	90.5 ± 17.6	87.1 ± 15.0	<0.001	0.062
FEV_1_/FVC ratio, %	80.6 ± 16.1	75.0 ± 11.5	74.1 ± 8.0	80.0 ± 8.2	71.5 ± 15.3	0.044	0.017
Blood eosinophils, %	2.0 ± 0.9	3.8 ± 6.0	5.6 ± 4.3	4.6 ± 9.5	2.5 ± 1.9	0.485	0.307
Sputum eosinophils, %	0.4 ± 0.8	1.9 ± 2.2	1.5 ± 1.3	2.1 ± 1.5	2.3 ± 3.2	<0.001	0.417
FeNO, ppb	16.1 ± 10.6	27.3 ± 27.6	34.3 ± 33.1	23.6 ± 24.5	23.4 ± 23.3	0.002	0.229
Anxiety and depression							
SAS score	31.8 ± 7.2	42.1 ± 10.8	42.0 ± 10.8	43.3 ± 9.8	41.2 ± 11.9	<0.001	0.763
SDS score	34.0 ± 11.4	43.8 ± 11.0	44.9 ± 11.3	44.9 ± 8.7	41.6 ± 12.4	<0.001	0.416

Plus–minus values are mean ± SD. Continuous data were compared with the use of the *t* test and One Way ANOVA, and categorical data were compared with the use of Pearson’s Chi square test. *CA* classic asthma, *CVA* cough-variant asthma, *CTVA* chest tightness variant asthma. The body-mass index (BMI) is the weight in kilograms divided by the square of the height in meters. *FEV*_*1*_ forced expiratory volume in 1 s, *FVC* forced vital capacity, *FeNO* fractional exhaled nitric-oxide, *SAS* self-rating anxiety scale, *SDS* self-rating depression scale

### Clinical features in CA, CVA, and CTVA

Patients with CA, CVA, and CTVA were similar in levels of serum total IgE. Thirteen of the CA cases (43.3%), ten of the CVA cases (37.0%), and seven of the CTVA (23.3%) cases have a history of atopy; the prevalence of atopy was not significant different among three asthmatic groups.

Then we investigated pulmonary function and bronchial responsiveness for patients with CA, CVA, and CTVA. FEV_1_ (% Predicted) in CA, CVA, and CTVA were 80.7 ± 14.6, 90.5 ± 17.6, and 84.2 ± 21.8%, respectively, with no significant differences. FEV_1_/FVC ratio (%) in CA, CVA, and CTVA were 74.1 ± 8.0, 80.0 ± 8.2, and 71.5 ± 15.3%, respectively. As compared with CA and CTVA, those with CVA had a higher FEV_1_/FVC ratio (%) (*P* = 0.017). Positive bronchial provocation test (BPT) in CA, CVA, and CTVA were 28 (93.3%), 22 (81.5%), and 28 (93.3%), respectively; and positive bronchial dilation test (BDT) in CA, CVA, and CTVA were 2 (6.7%), 5 (18.5%), and 2 (6.7%), respectively. There was no significant difference in the results of the bronchial challenge test and bronchodilator test among the three groups.

Recently, numerous studies have demonstrated that anxiety and depression are common and relevant comorbidities in asthmatic patients [15–17]. In this study, to investigate the correlation among different presentations of asthma and psychiatric disorders, all patients were evaluated with SAS and SDS questionnaires. The results showed no significant differences among patients with CA, CVA, and CTVA, in terms of SAS score, or SDS score.

### Induced sputum eosinophil count and FeNO in CA, CVA, and CTVA

It is well established that asthma is a chronic inflammatory airway disease, in which eosinophils play a central role [18, 19]. The mean eosinophil percentages in induced sputum in patients with CA, CVA, and CTVA were 1.5 ± 1.3, 2.1 ± 1.5, and 2.3 ± 3.2%, respectively, which were all significantly higher (*P* < 0.01) than that of the control group (0.4 ± 0.85%) (Fig. 1b). However, no significant difference in eosinophil percentages was found among three asthmatic groups. FeNO in patients with CA, CVA, and CTVA were 34.3 ± 33.1, 23.6 ± 24.5, and 23.4 ± 23.3%, respectively. A higher level of FeNO was observed in patients with CA when compared with health controls (16.1 ± 10.6%), but not for the CVA or CTVA cases (Fig. 1c). There were no statistical differences in FeNO among patients with CA, CVA, and CTVA.

### Proteomic measurements in induced sputum in CA, CVA, and CTVA

**Fig. 2:**
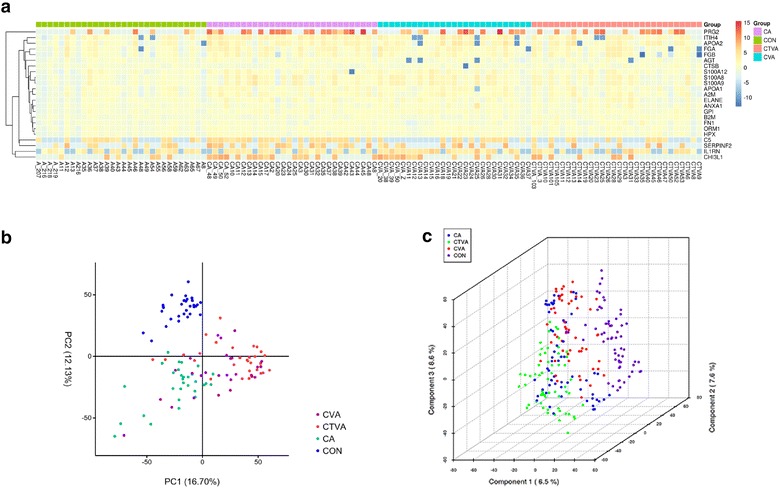
Sputum proteomics data from healthy controls and asthma patients. Heat map of selected protein expression in induced sputum of asthma patients and health controls. Expression patterns in selected proteins are visually presented as expression matrix using a relative scale ranging from -10 (*blue*) to 15 (*red*) (**a**). Principal components analysis (PCA) of protein expression in three asthmatic patients and matched-control subjects. CON, *blue*; CA, *light green*; CVA, *purple*; CTVA, *red* (**b**). *CA* classic asthma, *CVA* cough-variant asthma, *CTVA* chest tightness variant asthma

To further evaluate the difference of airway inflammation among patients with CA, CVA, and CTVA, proteins were measured in induced sputum. In the heat map, the selected protein expression was mostly increased in the CA, CVA, and CTVA groups as compared with controls (Fig. 2a). PCA of protein expression was also used to assess the major sources of variation among samples. Most of the samples were classified into two groups, indicating that the constituents of the samples from patients and controls were significantly different. In this study, PC1 described 16.7% of the variance and PC2 accounted for an additional 12.1% of the variation (Fig. 2b). Similar results were obtained form PLS-DA analysis (Fig. 2c).

**Fig. 3:**
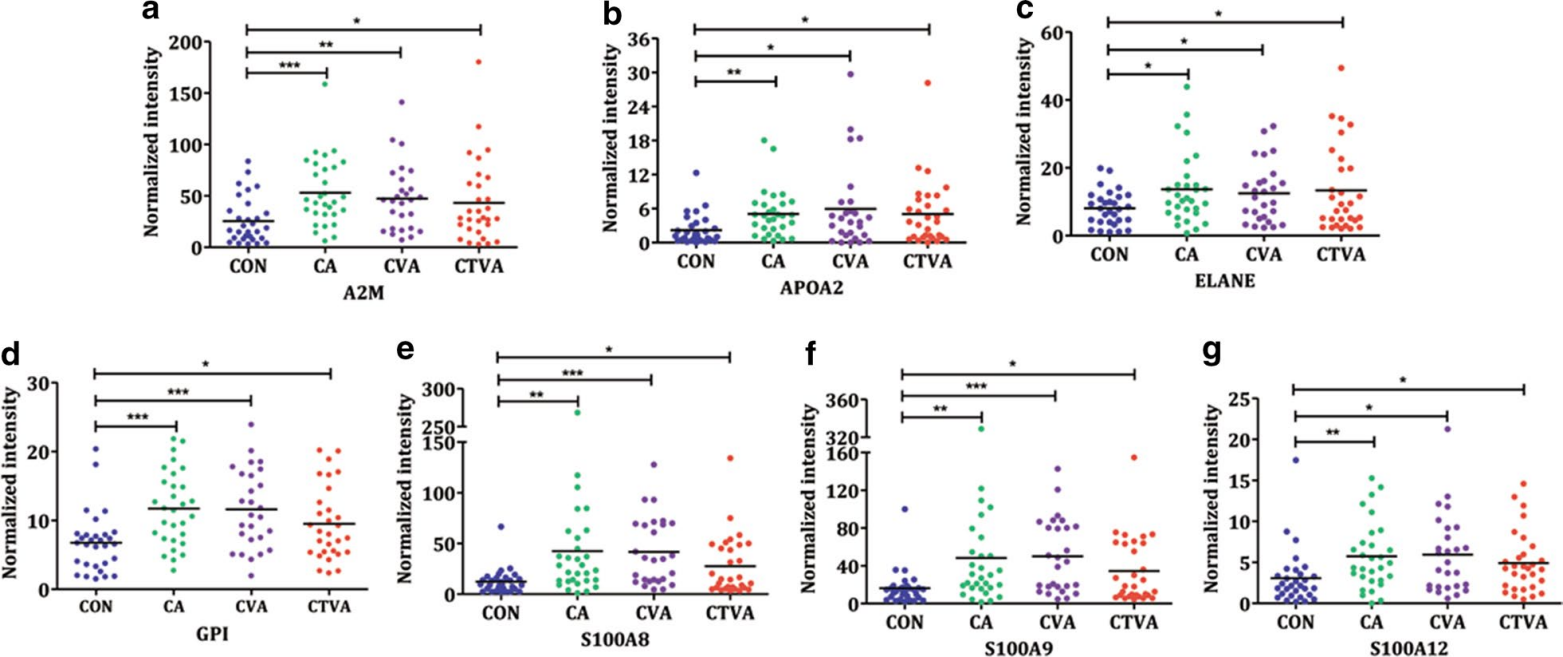
High expression of secreted proteins in three asthmatic patients. A2 M (**a**), APOA2 (**b**), ELANE (**c**), GPI (**d**), S100A8 (**e**), S100A9 (**f**), and S100A12 (**g**). Mean values are represented as *horizontal bars*. *P < 0.05, **P < 0.01, ***P < 0.001; *CA* classic asthma, *CVA* cough-variant asthma, *CTVA* chest tightness variant asthma

Although 1126 proteins were identified in the sputum from patients with CA, CVA, and CTVA (Additional file 1: Table S1), the levels of most of proteins was similar between three groups. Significant elevation of 23 proteins was observed in asthmatic sputum as compared with controls (Table 2). These proteins are involved in multiple biological process, including immunity, inflammatory, chemokines, protease, protease inhibitor, metabolism, transport, hydrolase, and vasculogenesis. Compared with healthy controls, we observed a significant increase of A2 M, APOA2, ELANE, GPI, S100A8, S100A9, and S100A12 in all three asthmatic groups (Fig. 3). In addition, distinctive biomarkers in sputum were also found: (1) CA: AGT, APOA1, C5, CHI3L1, FGA, FGB, HPX, ITIH4, ORM1, PRG2, and SERPINF2; (2) CVA: APOA1, CHI3L1, FGB, HPX, IL1RN, ITIH4, and ORM1; (3) CTVA: ANXA1, B2 M, CTSB, FN1, and PRG2 (Additional file 2: Figure S1).

**Table 2:** The list of proteins significant elevated in induced sputum of asthmatic patients as compared with controls (data were showed as fold change of the controls)

	Biological process or molecular function	CA	CVA	CTVA	Correlation with eosinophils	
					r	*P*
A2M	Protease inhibitor	2.1	1.9	1.7	0.0542	0.5614
ANXA1	Adaptive immunity, immunity, inflammatory response	1.4	2.3	1.7	−0.0775	0.4063
APOA2	Host-virus interaction, lipid transport, transport	2.3	2.7	2.3	0.0370	0.6918
ELANE	Hydrolase, protease, serine protease	1.6	1.5	1.6	0.1145	0.2191
GPI	Angiogenesis, gluconeogenesis, glycolysis	1.7	1.7	1.4	0.2537	0.0058
S100A8	Apoptosis, autophagy, chemotaxis, immunity, inflammatory	3.5	3.4	2.3	0.1505	0.1053
S100A9	Apoptosis, autophagy, chemotaxis, immunity, inflammatory	3.0	3.1	2.1	0.1768	0.0565
S100A12	Immunity, inflammatory response, innate immunity	1.9	2.0	1.6	0.2929	0.0014
AGT	Vasoactive, vasoconstrictor	1.4	NS	NS	0.0971	0.2979
C5	Complement pathway, cytolysis, immunity, inflammatory	2.1	NS	NS	−0.0420	0.6526
FGA	Blood coagulation, hemostasis, immunity	1.9	NS	NS	0.0289	0.7573
SERPINF2	Serine protease inhibitor	2.6	NS	NS		
APOA1	Metabolism, transport	1.7	1.6	NS	0.0495	0.5960
CHI3L1	Apoptosis, inflammatory response	4.4	3.4	NS	−0.0058	0.9497
FGB	Blood coagulation, hemostasis, immunity	1.7	1.8	NS	0.0529	0.5709
HPX	Host-virus interaction, transport	1.6	1.3	NS	0.1797	0.0525
ITIH4	Inflammatory responses to trauma	2.5	1.9	NS	−0.0187	0.8416
ORM1	Transport protein in the blood stream	1.4	1.4	NS	0.1806	0.0514
IL1RN	Immune response	NS	3.1	NS	0.1631	0.0789
PRG2	Hydrolase	2.0	NS	7.0	−0.0388	0.6777
B2M	Immunity	NS	NS	1.6	0.0976	0.2994
CTSB	Hydrolase, protease, thiol protease	NS	NS	1.4	0.0662	0.4786
FN1	Acute phase, angiogenesis, cell adhesion, cell shape	NS	NS	1.6	0.0487	0.6021

*NS* not significant

## Discussion

The patients we recruited with CA, CVA, and CTVA had similar clinical and inflammatory characteristics with no differences with respect to atopy, serum IgE, FEV_1_ (% Predicted), sputum eosinophils, and FeNO. In these three groups of asthma types, we found a similar proteomic pattern with minor differences in induced-sputum supernatants. Therefore, although patients with CA, CVA, and CTVA are diagnosed on the basis of different presenting symptoms, our findings support the contention that the inflammatory mechanisms of eosinophilic inflammation are similar in CA, CVA and CTVA.

In the present study, sputum eosinophil percentages were significantly higher in patients with CA, CVA, and CTVA than that of controls. Increased sputum and tissue eosinophil levels have been demonstrated in the patients with CA [20–22]. In the past decades, numbers of studies were conducted to investigate the nature of inflammation in the airways of CVA patients. It has been reported that eosinophil count in induced sputum, in bronchoalveolar lavage or in biopsy specimens are elevated in patients with CVA [23–25]. We also found eosinophilic inflammation in CA and CVA, and also for the first time in the newly-described asthmatic condition of CTVA sputum eosinophilia [9]. Thus these three types of asthma conditions are characterized by eosinophilia.

One of the aims of the present study was to compare the degree of airway inflammation among patients with CA, CVA, and CTVA. However, a comparison of the sputum eosinophil measurements in these three groups showed no significant differences. FeNO is validated, commonly used as a noninvasive marker of airway inflammation in asthma [26]. FeNO measurements are increasingly applied for diagnosis and monitoring of asthma, without the practical difficulties associated with bronchial biopsy or sputum induction [27]. In this study, we did not observe a significant difference in FeNO among patients with CA, CVA, and CTVA, which further implied that airway inflammation may not be causally related to differences in the presenting clinical manifestations of asthma.

Asthma is the manifestation of multitude reactions of biological, cellular and immunological events, in which cytokines, chemokines, and inflammatory pathways associated with T helper type 2 (T(H)2)-driven adaptive immunity have been demonstrated to play a central role in its pathogenesis [28, 29]. In order to identify novel biomarkers for asthma, MS was employed. More than a thousand proteins were revealed from the induced-sputum with the levels of most proteins being similar between different groups, suggesting a very good quality control. Over 20 proteins were found elevated in sputum from asthma patients. These proteins are involved in multiple biological process, including immunity, inflammatory, chemokines, protease, protease inhibitor, metabolism, transport, hydrolase, and vasculogenesis etc. We observed a significant increase of A2M, ANXA1, APOA2, ELANE, GPI, S100A8, S100A9, and S100A12 in all three asthmatic groups, among which S100A8, S100A9, and S100A12 were previously-reported biomarkers for asthma (Table 2). Next, we also observed that some proteins were expressed differentially in sputum from patients with various clinical presentations, for example: (1) increase in CA: AGT, APOA1, C5, CHI3L1, FGA, FGB, HPX, ITIH4, ORM1, PRG2, and SERPINF2; (2) increase in CVA: APOA1, CHI3L1, FGB, HPX, IL1RN, ITIH4, and ORM1; (3) increase in CTVA: B2M, CTSB, FN1, and PRG2 (Table 2).

In this study, we found eosinophilic inflammation in CA and CVA, and for the first time in the newly-described asthmatic condition of CTVA sputum eosinophilia. Thus these three types of asthma conditions are characterized by eosinophilia. We therefore analyzed the correlation of these newly-identified biomarkers with eosinophil counts. Interestingly, GPI and S100A12 were found to be highly associated with sputum eosinophil counts, suggesting a TH2 dependent response. Thus, this finding provides a rationale for CVA and CTVA therapies as those for CA, in the use of inhaled corticosteroids, and other potential targeted therapies for the Th2 pathway [30]. As specific biologic agents are developed, asthma-relevant cytokines or chemokines have been targeted in a number of ways. In the present study, several disease-special proteins were found in patients with CA, CVA, and CTVA, which may be used as disease biomarkers and therapeutic targets.

Several limitations of this study are worth discussing. First, the findings from our study should be confirmed by other studies. Future studies were warranted to validate our findings. Second, the induced-sputum sample was used in our study, which is secreted from airways, not link to gene or something else. Thus, it may not available to conduct some further analyses such as pathway analysis. Third, most proteins in three asthmatic group were similar. Thus, we can only look for differentiating differences, while failed to mention as a total asthma group.

## Conclusions

In summary, this study show that, in patients with CA, CVA, and CTVA who have similar clinical and inflammatory characteristics in terms of eosinophilic airway inflammation, there were no dramatic differences in the spectrum and levels of proteins in sputum supernatants. These findings support the concept that these three different presentations of asthma have similar pathogenetic mechanism in terms of an enhanced Th2 associated with eosinophilia. A common therapeutic strategy might be suggested for these different presentations of asthma. This study, for the first time, performed a proteomic analysis of the sputum supernatants in different presentations of asthmatic patients. In our MS analysis of sputum samples, more than 1000 proteins were identified, among which 23 secreted proteins were higher in patients than that in controls. Differential proteins involved in immunity, inflammatory, and chemokines were analyzed. Importantly, we identified a pool of novel biomarkers for diagnosis of asthma and to label its subtypes.

## Additional files

**Additional file 1: Table S1.** The proteins were identified in the sputum from patients with CA, CVA, and CTVA.

**Additional file 2: Figure S1.** High expression of secreted protein in CA, CVA, or CTVA patients. AGT (A), ANXA1 (B), APOA1 (C), B2M (D), C5 (E), CHI3L1 (F), CTSB (G), FGA (H), FGB (I), FN1 (J), HPX (K), IL1RN (L), ITIH4 (M), ORM1 (N), PRG2 (O), and SERPINF (P). Mean values are represented as horizontal bars. *P < 0.05, **P < 0.01, ***P < 0.001; CA, classic asthma; CVA, cough-variant asthma; CTVA, chest tightness variant asthma.

## Abbreviations

BMI: body-mass index
BPT: bronchial provocation test
BDT: bronchial dilation test
CA: classic asthma
CVA: cough-variant asthma
CTVA: chest tightness variant asthma
FEV_1_: forced expiratory volume in 1 s
FVC: forced vital capacity
FeNO: fractional exhaled nitric-oxide
PCA: principal components analysis
PEF: peak expiratory flow
PBS: phosphate-buffered saline
SAS: self-rating anxiety scale
SDS: self-rating depression scale
PLS-DA: partial least squares discrimination analysis
