# Supplementary material for: Proteomic analysis of sputum reveals novel biomarkers for various presentations of asthma
Source: J Transl Med. 2017 Aug 4;15:171. doi: 10.1186/s12967-017-1264-y (PMC5544989; doi:10.1186/s12967-017-1264-y)

**FIGURE LEGENDS**

**Supplement Figure 1.** High expression of secreted protein in CA, CVA, or CTVA patients. AGT (A), ANXA1 (B), APOA1 (C), B2M (D), C5 (E), CHI3L1 (F), CTSB (G), FGA (H), FGB (I), FN1 (J), HPX (K), IL1RN (L), ITIH4 (M), ORM1 (N), PRG2 (O), and SERPINF (P). Mean values are represented as horizontal bars. **P* < 0.05, ***P* < 0.01, ****P* < 0.001; CA, classic asthma; CVA, cough-variant asthma; CTVA, chest tightness variant asthma.


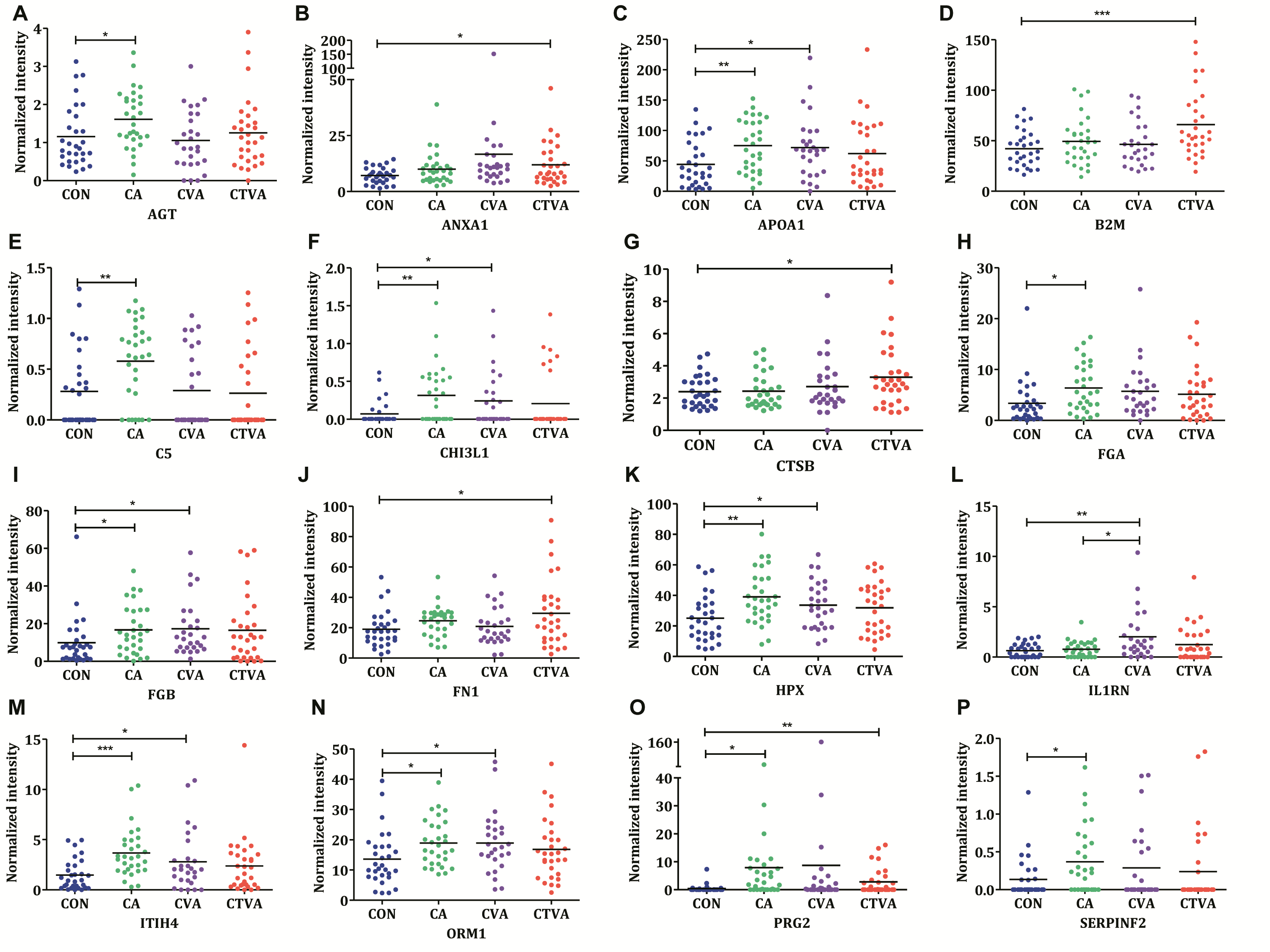

Supplement: Supplementary file 2 — Additional file 2: Figure S1. High expression of secreted protein in CA, CVA, or CTVA patients. AGT (A), ANXA1 (B), APOA1 (C), B2M (D), C5 (E), CHI3L1 (F), CTSB (G), FGA (H), FGB (I), FN1 (J), HPX (K), IL1RN (L), ITIH4 (M), ORM1 (N), PRG2 (O), and SERPINF (P). Mean values are represented as horizontal bars. *P < 0.05, **P < 0.01, ***P < 0.001; CA, classic asthma; CVA, cough-variant asthma; CTVA, chest tightness variant asthma. [file 12967_2017_1264_MOESM2_ESM.doc]
